# Caspase-1 has a critical role in blood-brain barrier injury and its inhibition contributes to multifaceted repair

**DOI:** 10.1186/s12974-020-01927-w

**Published:** 2020-09-09

**Authors:** Hila Israelov, Orly Ravid, Dana Atrakchi, Daniel Rand, Shirin Elhaik, Yael Bresler, Rachel Twitto-Greenberg, Liora Omesi, Sigal Liraz-Zaltsman, Fabien Gosselet, Michal Schnaider Beeri, Itzik Cooper

**Affiliations:** 1 The Joseph Sagol Neuroscience Center, Sheba Medical Center, 52621 Tel Hashomer, Ramat Gan Israel; 2 Sackler Faculty of Medicine, Tel-Aviv University, Tel Aviv, Israel; 3 Department of Pharmacology, The Institute for Drug Research, The Hebrew University of Jerusalem, Jerusalem, Israel; 4 Institute for Health and Medical Professions, Department of Sports Therapy, Ono Academic College, Kiryat Ono, Israel; 5 UR 2465, Blood-brain barrier Laboratory (LBHE), Artois University, F-62300 Lens, France; 6 School of Psychology, Interdisciplinary Center (IDC), Herzliya, Israel; 7 Department of Psychiatry, The Icahn School of Medicine at Mount Sinai, New York, NY 10029 USA; 8 The Nehemia Rubin Excellence in Biomedical Research – The TELEM Program, Sheba Medical Center, Tel-Hashomer, Israel

**Keywords:** Caspase-1, Inflammasome, Blood-brain barrier, Paraoxon, Permeability, Adhesion, Transmigration, Inflammation

## Abstract

**Background:**

Excessive inflammation might activate and injure the blood-brain barrier (BBB), a common feature of many central nervous system (CNS) disorders. We previously developed an in vitro BBB injury model in which the organophosphate paraoxon (PX) affects the BBB endothelium by attenuating junctional protein expression leading to weakened barrier integrity. The objective of this study was to investigate the inflammatory cellular response at the BBB to elucidate critical pathways that might lead to effective treatment in CNS pathologies in which the BBB is compromised. We hypothesized that caspase-1, a core component of the inflammasome complex, might have important role in BBB function since accumulating evidence indicates its involvement in brain inflammation and pathophysiology.

**Methods:**

An in vitro human BBB model was employed to investigate BBB functions related to inflammation, primarily adhesion and transmigration of peripheral blood mononuclear cells (PBMCs). Caspase-1 pathway was studied by measurements of its activation state and its role in PBMCs adhesion, transmigration, and BBB permeability were investigated using the specific caspase-1 inhibitor, VX-765. Expression level of adhesion and junctional molecules and the secretion of pro-inflammatory cytokines were measured in vitro and in vivo at the BBB endothelium after exposure to PX. The potential repair effect of blocking caspase-1 and downstream molecules was evaluated by immunocytochemistry, ELISA, and Nanostring technology.

**Results:**

PX affected the BBB in vitro by elevating the expression of the adhesion molecules E-selectin and ICAM-1 leading to increased adhesion of PBMCs to endothelial monolayer, followed by elevated transendothelial-migration which was ICAM-1 and LFA-1 dependent. Blocking caspase-8 and 9 rescued the viability of the endothelial cells but not the elevated transmigration of PBMCs. Inhibition of caspase-1, on the other hand, robustly restored all of barrier insults tested including PBMCs adhesion and transmigration, permeability, and VE-cadherin protein levels. The in vitro inflammatory response induced by PX and the role of caspase-1 in BBB injury were corroborated in vivo in isolated blood vessels from hippocampi of mice exposed to PX and treated with VX-765.

**Conclusions:**

These results shed light on the important role of caspase-1 in BBB insult in general and specifically in the inflamed endothelium, and suggest therapeutic potential for various CNS disorders, by targeting caspase-1 in the injured BBB.

## Introduction

There is growing evidence indicating that blood-brain barrier (BBB) dysfunction might be involved in the pathogenesis of neurodegenerative diseases and that it predicts poor neurological outcomes [1–3]. The BBB is a selective barrier formed by the endothelial cells (ECs) lining cerebral capillaries, whose properties are created and maintained by perivascular elements such as the closely associated astrocytic end-feet processes and pericytes [4]. Functionally, the BBB protects the brain from fluctuations of the circulation and plays a major role in maintaining the homeostatic environment required for normal brain function by forming a selective and dynamic barrier. Under normal physiological conditions, the presence of continuous tight junctions (TJ) between adjacent ECs of brain capillaries forms an impermeable seal that significantly prevents transport of polar solutes, macromolecules, and cells from the circulation into the brain [5]. BBB disruption may be manifested as a leakage through the brain endothelium by opening of intercellular TJ (i.e., the paracellular pathway) or by the disrupted function of the transcellular pathway, meaning vesicular transcytosis malfunctioning of various transporters, receptors, and efflux pumps [6]. Under healthy physiological conditions, very few immune cells penetrate the BBB for the purpose of immune surveillance. However, in various pathological conditions, the barrier becomes compromised, resulting in infiltration of immune cells to CNS parenchyma, whose subsequent activity appears to underlie the onset and progression of CNS diseases [7]. The inflammatory response is a double-edged sword; it is beneficial and essential in maintaining homeostasis, but it has the potential to cause tissue damage and deleterious effects when over activated or dysregulated [8].

Trans-endothelial migration of leukocytes from the circulation to the CNS parenchyma is a multistep process, depending on the interaction of adhesion molecules on the leukocyte surface with corresponding ligands expressed on ECs in response to infections or inflammatory insult [9]. The initial contact of leukocytes with ECs is mediated by selectins participating in the early tethering and rolling of leukocytes on the endothelial surface. Integrins, then, establish a stronger molecular interaction that results in arrest and spreading of leukocytes on the endothelial surface (diapedesis). When leukocytes roll along the endothelium, their integrins become activated following cell contact with inflammatory stimuli that are expressed on or secreted by the ECs, such as the chemokines interleukin-8 (IL-8) and monocyte chemoattractant protein 1 (MCP-1) [10, 11]. In particular, they bind to members of the immunoglobulin gene superfamily, intercellular adhesion molecules 1 and 2 (ICAM-1 and ICAM-2), and vascular cell adhesion molecule 1 (VCAM-1) [9]. Finally, the leukocytes migrate through the endothelium and the remainder of the blood vessel wall, directly to the inflammatory site. It is likely that factors contributing to the circumvention of the normal restrictive properties of the BBB by immune cells are operating on several steps of this process [9].

Malfunctioning BBB might be the result of numerous insults including exposure to chemicals, viral and bacterial infection, and acute conditions such as stroke and traumatic brain injury [12–15]. Organophosphates (OPs) chemical exposure has been linked to neurological abnormalities found in war veterans and in individuals in occupational settings where exposure to OPs is present. We speculate that exposure to OPs may underlie some prolonged “unexplained” neurological pathologies, through ongoing inflammatory subtle BBB disruption leading to malfunction of neurochemical gentle homeostasis regulation.

Caspase-1 is a core component of the inflammasome complexes [16] which regulate activation, production, and secretion of the pro-inflammatory cytokines IL-1β and IL-18 [17]. Importantly, recent studies suggest that inflammasomes are involved in many neurodegenerative and cerebrovascular diseases [18, 19]. Our study attempted to identify inflammation-inducing factors at the BBB, by examining several adhesion molecules, cytokines, and chemokines whose levels were modulated in the BBB endothelium after exposure to OPs, with a focus on caspase-1 crucial role in these processes.

The importance of caspase-1 has been recently shown in various CNS disorders and specifically in Alzheimer’s disease (AD) in which BBB dysfunction plays a major role. In mouse models, knocking out caspase-1, resulted in protection from memory deficits and improved Aβ clearance [20, 21]. Treating AD-like mice with VX-765—a caspase-1 selective inhibitor—led to better cognition and less AD-like pathology [20]. In humans, substantially increased amounts of cleaved (active) caspase-1 fragments were observed in brains from AD patients compared to controls, consistent with chronic inflammasome activation. Linkage between inflammasome activation and brain exposure to chemicals has also been reported; exposure to the chemical herbicide paraquat caused elevated brain inflammation in mice, demonstrated by increased levels of activated caspase-1 and mature IL-1β in the hippocampus [22] and specifically, OPs have been shown to elevate caspase-1 levels in non-cerebral cells [23]. Interestingly, activation of inflammasomes markedly decreased the expression of tight and adherens junction proteins in non-cerebral ECs [20, 24, 25]. These findings promoted our interest in examining the role of the inflammasome, and particularly of caspase-1, in mediating leukocytes transmigration and hindering BBB integrity following OPs exposure.

Our previous study [26] demonstrated that the OP paraoxon (PX) directly affects the BBB in vitro. PX attenuated viability, integrity, and junctional mRNA and protein expression levels of brain-like endothelial cells (BLECs). This led us to hypothesize that harmful substances such as PX might also induce an inflammatory reaction that leads to the transmigration of immune cells through the BBB which may be dependent on caspase-1 activation. In the present work, we demonstrated that PX induces increased adhesion and transmigration of peripheral blood mononuclear cells (PBMCs) to and through the BBB. Our in vitro and in vivo results further indicate that this phenomenon is dependent on caspase-1 activation and shed light on important BBB-related cellular immune responses that may be relevant to prevention of neurodegenerative processes.

## Material and methods

### Materials

Paraoxon-ethyl was purchased from Sigma. For assessing the BBB response, PX was freshly prepared in ethanol to a 400-mM stock solution, and immediately diluted in the cell medium to the desired final concentrations. VX-765, was purchased from ApexBio (#A8238). BCECF (#B-1170) from molecular probes was used for fluorescence labeling of the PBMCs in adhesion and transmigration assay. Mouse anti-ICAM-1/CD54 antibody (#BBA3, R&D SYSTEMS) and mouse anti-E-selectin/CD62E antibody (#MAB2150, MERCK) were used for the in-cell western blot experiments and for immunocytochemistry. This anti-ICAM-1 antibody was also used as a neutralizing antibody in transmigration assay. IR-conjugated secondary antibodies were purchased from LI-COR Biosciences; IRDye 800cw (#926-32212) and CellTag700 (#926-41090). Lifitegrast, an LFA-1 antagonist, was obtained from Cayman chemical (#22588). Human recombinant IL-1β (cyt-208) and Human IL-18 (cyt-269) were purchased from Prospec (Rehovot, Israel). Caspase-8 inhibitor Z-LEHD-FMK (#4805-510), and Caspase-9 inhibitor Z-IETD-FMK (#4810-510) were purchased from MBL Life science. Neutralizing IgG monoclonal antibody against human IL-1β (#MAB-201) and IL-8 (#MAB-208) were obtained from R&D Systems, anti-hIL-18 (#D004-3) was obtained from MBL. Mouse anti-VE-cadherin antibody (#sc-9989) was obtained from Santa Cruz Biotechnology and was used for immunocytochemistry. Goat anti-mouse Alexa Fluor-488 secondary antibody was purchased from Invitrogen (A-11001). Fast SYBR™ Green (#4385601) was purchased from ThermoFisher.

### Media

BLECs and pericytes were grown in endothelial cell medium (ECM) (Sciencell) supplemented with 5% fetal calf serum (Gibco), ECGS supplements, and 50 μg/ml gentamycin (Biological industries). In order to induce brain-like phenotype, ECs monolayers were grown in medium containing 2/3 ECM and 1/3 pericytes-conditioned medium (ECM medium collected after 3 days culture with pericytes), as previously published [26, 27].

### BBB in vitro model

To investigate the cellular response to chemical injury, a human BBB model which is well characterized for studying BBB injury, inflammation, and barrier functionality was utilized [26, 28]. The generation of these ECs relies on biological principles observed in the repair of BBB in the human body. The in vivo repair of the endothelium is mediated by endothelial progenitor cells that migrate to the sites of endothelial injury, incorporate in the growing vessel and differentiate into ECs [29, 30]. The human BBB model was generated using human cord blood-derived hematopoietic stem cells; CD34+ cells were isolated from umbilical cord blood. Parents of infants signed a consent form. All protocols were done with the authorization of the French Ministry of Higher Education and Research (CODECOH Number DC2011-1321). CD34+ cells were initially differentiated into ECs followed by the induction of BBB properties by co-culture with bovine brain pericytes on matrigel-coated insert (Transwell®, 5 μm). The produced BLECs express TJ proteins and transporters typically observed in brain endothelium and maintain expression of most in vivo BBB properties for at least 20 days [28]. We used the co-culture system or monoculture of CD34+ ECs grown in PCM. BBB injury was generated by exposure to the organophosphate PX, as previously characterized [26].

### Cell death by LDH release

The toxicity of PX was investigated on BLECs monolayers that were seeded in 96-well plates as well as on BLECs in Transwells experiments using a cytotoxicity detection kit (Promega, Madison, WI, USA). An aliquot of 50 μl was taken to quantify the amount of lactate dehydrogenase (LDH) released from the cells as a consequence of cell membrane rapture. The test was performed according to the manufacturer’s instructions and absorption was measured at 492 nm by an ELISA plate reader (Tecan, Switzerland).

### Viability assay by PrestoBlue

The viability of BLECs monolayers was assessed using PrestoBlue cell viability reagent (#XA13261, Invitrogen), according to the manufacturer’s protocol. The BLECs were cultured in gelatin-coated 96-well plates with Prestoblue (diluted 1:10 in ECM) for 10 min. The fluorescence was measured at excitation/emission of 560/590 nm by spectra max Gemini plate reader (Molecular Devices).

### PBMCs isolation

PBMCs include the major subpopulation of migrating leukocytes: monocytes and lymphocytes. Therefore, we have chosen to employ this population of immune cells in our adhesion and transmigration assays [31]. Leukocyte-enriched blood was purchased from blood bank (MADA) for research. The blood was diluted 1:1 with Hanks’ balanced salt solution (HBSS, #H6648, Sigma), 4 ml of the diluted blood was added to 3 ml of Ficoll (Ficoll paque plus, #17144002, Sigma). The tubes were centrifuged for 30 min (400 g, 20 °C) to separate the blood components. The fraction of white blood cells was gently collected, washed twice with phosphate-buffered saline (PBS) and centrifuged (10 min, 250 g, 20 °C). Red blood cell lysis buffer (#11814389001, Sigma) was added to the pellet (2:1) and pipetted for 1 min, incubated for 5 min at room temperature and centrifuged (5 min, 500 g, 20 °C). The remaining cells were washed and centrifuged (5 min, 500 g, 20°) twice. The remaining pellet consisted of the isolated PBMCs.

### PBMCs adhesion assay

PBMCs adhesion assay was conducted as previously described with some modifications [32]. BLECs were plated on 96-well black plates with clear bottom in 100 μl medium/well and were treated (or not) for 24 h with PX. PBMCs were fluorescently labeled as follows: 1 × 10^6^ cells/50 μl PBS loaded with 0.5 μl of 2′,7′-Bis(2-carboxyethyl)-5(6)-carboxyfluorescein acetoxymethyl ester (BCECF) for final concentration of 10 μM, for 30 min incubation at 37 °C. The fluorescently labeled PBMCs were washed twice and loaded onto the BLECs monolayers for 30 min of incubation in rotation at 37 °C (2 × 10^5^ cells in 50 μl ECM/well). Monolayers were gently washed 3 times with PBS by pipetting up and down to remove PBMCs that did not adhere firmly. The adhesion values were normalized to the cell viability values under each condition. Relative fluorescence of the attached PBMCs was acquired on an Infinite 200 PRO (Tecan) plate reader using the excitation/emission wavelength settings: 485/530 nm. For each experiment, a calibration curve was prepared from a serial dilution of a known amount of fluorescently labeled PBMCs.

### PBMCs transmigration assay

**Scheme 1:**
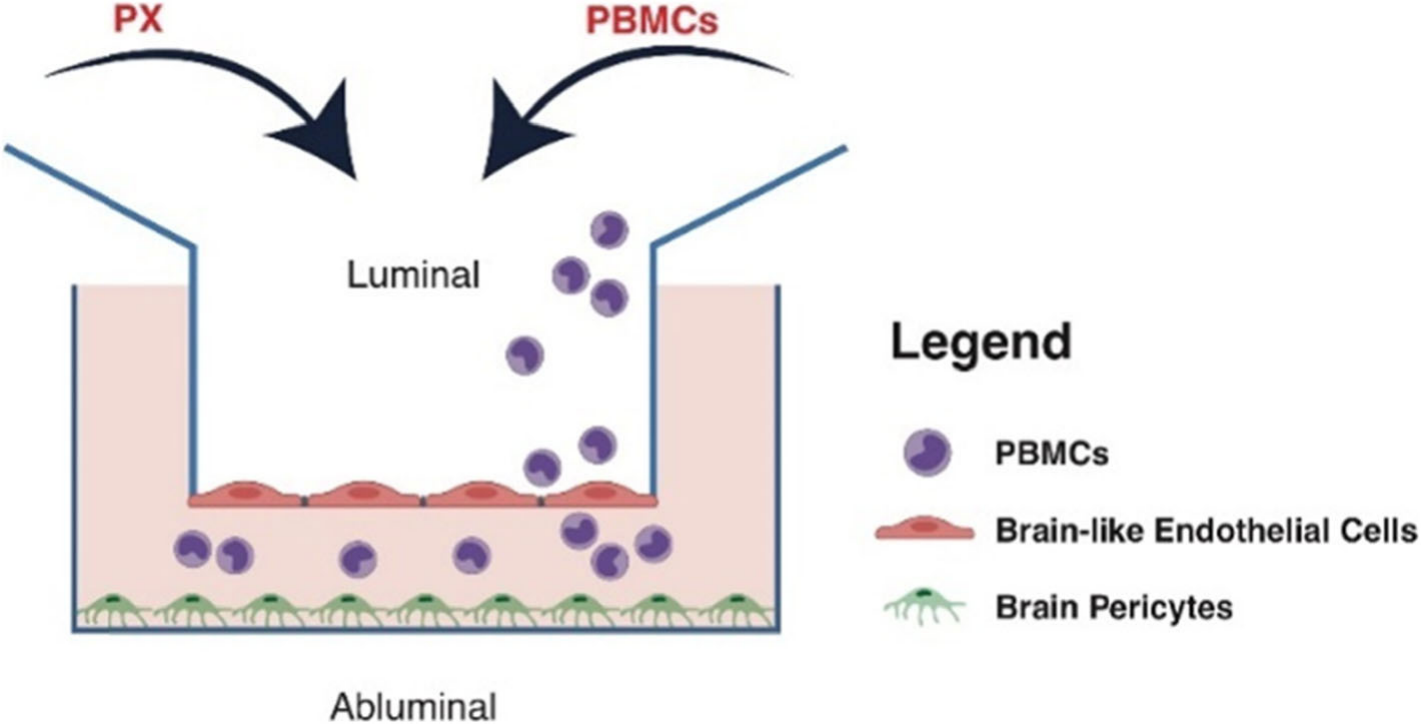
Schematics of the BBB in vitro model. The in vitro BBB co-culture system is composed of brain-like endothelial cells (BLECs) grown on matrigel-coated Transwell inserts, co-cultured with bovine brain pericytes grown at the abluminal compartment

The co-culture BBB in vitro model was applied for the transmigration assay. Brain bovine pericytes were seeded at a density of 1 × 10^4^/well on 24 well gelatin-coated plates (#3524, Costar) and cultured in ECM medium. Human CD34^+^-derived ECs were seeded at a density of 2 × 10^4^/insert onto the matrigel-coated (#356234, BD Biosciences) transwell inserts with a pore size of 5 μm (#3421, Costar). ECs were then co-cultured with the pericytes in a non-contact setting for 6-8 days to acquire BBB properties during this period. The inserts were treated (or not) with PX at the luminal and abluminal sides for 24 h, after which fluorescently labeled PBMCs were added onto the luminal (“blood”) side of each insert (2 × 10^5^/insert) for 4 h. The inserts were removed and relative fluorescence of the abluminal (“brain”) side was measured, using an Infinite 200 PRO (Tecan) plate reader, with the excitation/emission wavelength settings: 485/530 nm. The number of PBMCs that migrated trough the barrier was calculated using a standard curve. Since BCECF is a pH sensitive dye, we ruled out any PX/pH-related artifact by measuring the fluorescence of different amount of BCECF-labeled PBMCs in control and PX containing mediums and found that the PX containing medium itself had no influence on fluorescence intensity (Fig. S1, supplementary). Schematic representation of the transmigration assay and the in vitro BBB model is shown in Scheme 1.

### In cell Western blot assay

The protein expression levels of ICAM-1 and E-selectin were determined with an in-cell Western blot assay. BLECs monolayers were grown on gelatin-coated 96-well plates until reached full confluence and were treated (or not) with PX for 24 h. The BLECs were then fixed with 4% ice-cold paraformaldehyde in PBS for 15 min, washed and permeabilized with 0.1% Triton x100/PBS followed by 1 h incubation in blocking buffer (10% normal horse serum/0.1% Triton x100/PBS). Then, the cells were incubated with the primary antibodies (overnight, 4 °C); both were diluted to a concentration of 10 μg/ml, in blocking buffer. Following 3 washing steps, the cells were probed with appropriate secondary antibodies (IRDye 800cw) for 1 h. IRDye CELL-TAG700 was used for normalization of cell number, this dye accumulates inside the cells and provides a linear signal across cell numbers. The intensity of each well was quantified at both the 800 nm and 680 nm (for cell number) channels using the Odyssey (CLx, LI-COR Biosciences) analysis software. Results are presented as the normalized ratio of 800 nm to 680 nm signal for each well.

### Gene expression—rtPCR

BLECs monolayers were grown in 6 cm plates, the cells were expanded on gelatin-coated dishes in ECM medium (1/3 pericytes-conditioned) and treated with PX (or not) for 24 h when reached full confluence. RNA isolation was performed using the RNA Purification kit (NucleoSpin®) following the protocol of purification from cultured cells and tissues. The cDNA was synthesized using the qScript™ cDNA Synthesis kit (Quanta Bioscience™). To precisely quantify specific mRNA expression, RT-PCR Step One Plus system (#8024, Applied Biosystems) was used. The following PCR-program was performed: 20s 95 °C (initial denaturation); 95 °C for 15 s, 60 °C for 30 s, repeated 40 times (amplification); 95 °C for 15 s, 60 °C for 1 min, 95 °C for 15 s (melting curve). The PCR results were evaluated using the Applied Biosystems software.

Primers used: ICAM-1 fw 'GGCCTCAGTCAGTGTGA', rev 'AACCCCATTCAGCGTCA'; PECAM-1 fw 'GAGTATTACTGCACAGCCTTCA', rev 'AACCACTGCAATAAGTCCTTTC'; VCAM-1 fw 'GGGAAGATGGTCGTGATCCTT', rev 'TCTGGGGTGGTCTC GATTTTA'; Caspase-1 fw 'TGCCTGTTCCTGTGATGTGGAGGA', rev 'CAGTGGTGGGCATCTGCGCT'; GAPDH as housekeeping gene fw 'GGCCTCCAAGGAGTAAGACC', rev 'AGGGGTCTACATGGCAACTG'

### IL-8 secretion

Human Quantikine ELISA Kit (#D8000C, R&D Systems) was used for detection of the chemokine IL-8 in BLECs medium according to the manufacturer’s instructions. The output was measured at 450 nm, by absorbance plate reader (Tecan, Switzerland).

### Permeability assay

Filter inserts of 0.4 μm pore size (Corning, #3401), containing confluent monolayers of BLECs (treated or not with PX for 24 h), were placed in the 12-well plate (Costar) to acclimate for 1 h in HEPES-buffered Ringer’s solution (RHB), after which compound solution containing the fluorescent integrity marker sodium fluorescein (50 μg/ml; Sigma) was added to the luminal side, and then placed on a shaker at 37 °C. Every 10 min inserts were transferred to a new 12-well plate filled with RHB over a period of 40 min. Aliquots from the abluminal solution were taken from each time point and the fluorescence was quantified. Inserts without cells were tested in each permeability measurement. Fluorescence detection was carried out on an Infinite 200 PRO (Tecan) plate reader using the excitation/emission wavelength settings: 485/538 nm. Permeability coefficient (Pe) was obtained from the slope of the calculated clearance curve as previously described [33].

### Immunocytochemistry

BLECs monolayers were grown on gelatin-coated 96-well plates until reached full confluence and were treated (or not) with PX for 24 h. The BLECs were then fixed with 4% ice-cold paraformaldehyde in PBS for 15 min, washed, and incubated in blocking buffer (10% normal horse serum/0.1% triton/PBS) for 1.5 h. The BLECs were then incubated with anti-ICAM-1, anti E-selectin, or anti-VE-cadherin (overnight, 4 °C), washed with 0.1% Tween20/PBS and immunostained with Alexa Fluor-488 goat anti-mouse secondary antibody (for 1 h at room temperature). Images were taken with Incucyte (Essen Bioscience) with 20× objective and analysis was conducted with the Incucyte software (Green integrated intensity per image/phase area per image).

### Caspase-1 activation assay

In order to assess the presence of active caspase-1, BLECs monolayers were incubated with FAM-YVAD-fmk (FAM-FLICA^TM^, Immunochemistry Technologies, Bloomington, MN, USA), fluorochrome inhibitor of caspase-1 that covalently binds to the active caspase enzyme [34], following the manufacturer’s instructions. Briefly, control and PX-treated BLECs were incubated with FLICA reagent in ECM for 50 min at 37 °C (in CO_2_ incubator) to allow for binding of FLICA to activated caspase-1. BLECs were washed twice to allow any unbound reagent to diffuse out of the cells. Images were taken with Incucyte (Essen Bioscience) with 20× objective and analysis was conducted with the Incucyte software (Green integrated intensity per image/phase area per image).

### In vivo treatments

Experiments were carried out on 8–10-weeks-old C57BL/6 J male mice with an average weight of 25 ± 2 g. All experiments were approved by the Sheba Medical Center Ethics Committee (IACUC#1140-18-ANIM). Mice were maintained in a controlled animal facility at 18–22 °C and 40–60% humidity, with a photoperiod of 12 h dark/12 h light. Animals were allowed free access to water and food during all experiments.

Mice were randomly divided into four groups according to the treatments (described in details in Table S1, supplementary). PX-treated mice were injected intramuscularly with PX (0.45 mg/kg) followed by intraperitoneal (IP) injection of the antidotes atropine (1.5 mg/kg, Sigma #A-0257) and obidoxime (20 mg/kg, Sigma #51063). Control mice were IM injected once with 0.9% NaCl. VX-765 (100 mg/kg, InvivoGen #inh-vx765i) was administered IP, 1 h before PX injection. Mice were sacrificed by cervical dislocation 4 or 24 h after treatments.

### Blood vessels isolation from hippocampi

Cerebral blood vessels of mice were isolated according to Miguel Gama-Sosa’s purification protocol, with some adaptations [35]. Briefly, mice were sacrificed and their hippocampi were immediately dissected and frozen. Hippocampi of each mouse were gently homogenized in 4 ml of cold 18% w/v Dextran solution in PBS, containing the RNase inhibitors: RNA protect (Sigma, #R7397) and RNA secure (ThermoFisher, #AM7006) and the detergent NP-40 (ThermoFisher, #28324). Homogenization was performed using a Teflon pestle tissue homogenizer and overhead stirrer (10 strokes). Homogenization resulted in a thick homogenate of low density that was overlaid over 5 ml of Ficoll (1.077 g\ml) to form a single-step discontinuous gradient. Centrifugation was performed for 30 min at 400 g and 4 °C. Pellets were then resuspended, washed twice with PBS and the final pellet was suspended in 1 ml Trizol for RNA extraction.

### RNA extraction from blood vessels

RNA was isolated by phase separation first by adding chloroform to samples in Trizol, followed by centrifugation (13,300×*g*, 15 min, 4 °C). The aqueous phase, which contains the RNA, was transferred to a new tube, and RNA was then precipitated with isopropanol.

### Nanostring analysis

Isolated RNA was used for mRNA expression analysis on the nCounter based NanoString instrument. RNA of each sample (100 ng) was hybridized using the Custom Gene Expression Panel on the nCounter system (both NanoString Technologies, Seattle, Washington, USA), following the manufacturer’s recommendations. Absolute read counts were quantified by the nCounter digital analyzer. The panel was designed to quantitate 40 target genes, 4 housekeeping genes, and additional positive and negative controls. Target genes are mainly relevant to inflammation in cerebral blood vessels. For downstream analysis, absolute read counts of all panel genes were extracted from the nSolver software (NanoString Technologies). Target genes were normalized to 4 reference genes and fold changes and associated statistics were performed using the Prism 8.0 software. Fold change of each gene was calculated as the ratio of the average gene expression in the control group to that of the PX-treated groups.

### Data and statistical analysis

Results are expressed as the mean ± standard error of the mean, with at least three biological repeats unless mentioned otherwise. Comparison between two groups was done with student’s *t* test and for three groups or more, with one-way analysis of variance (ANOVA) with Tukey’s multiple comparison test for post hoc analyses. For in vivo Nanostring study, statistical analysis was performed using one-way ANOVA with Fisher’s LSD test. GraphPad Prism 8.0 software was used for all statistical analyses (GraphPad Software Inc., La Jolla, CA). Differences were considered significant at *P* values < 0.05.

## Results

### Dose dependent adhesion and transmigration of PBMCs across the in vitro BBB

**Fig. 1:**
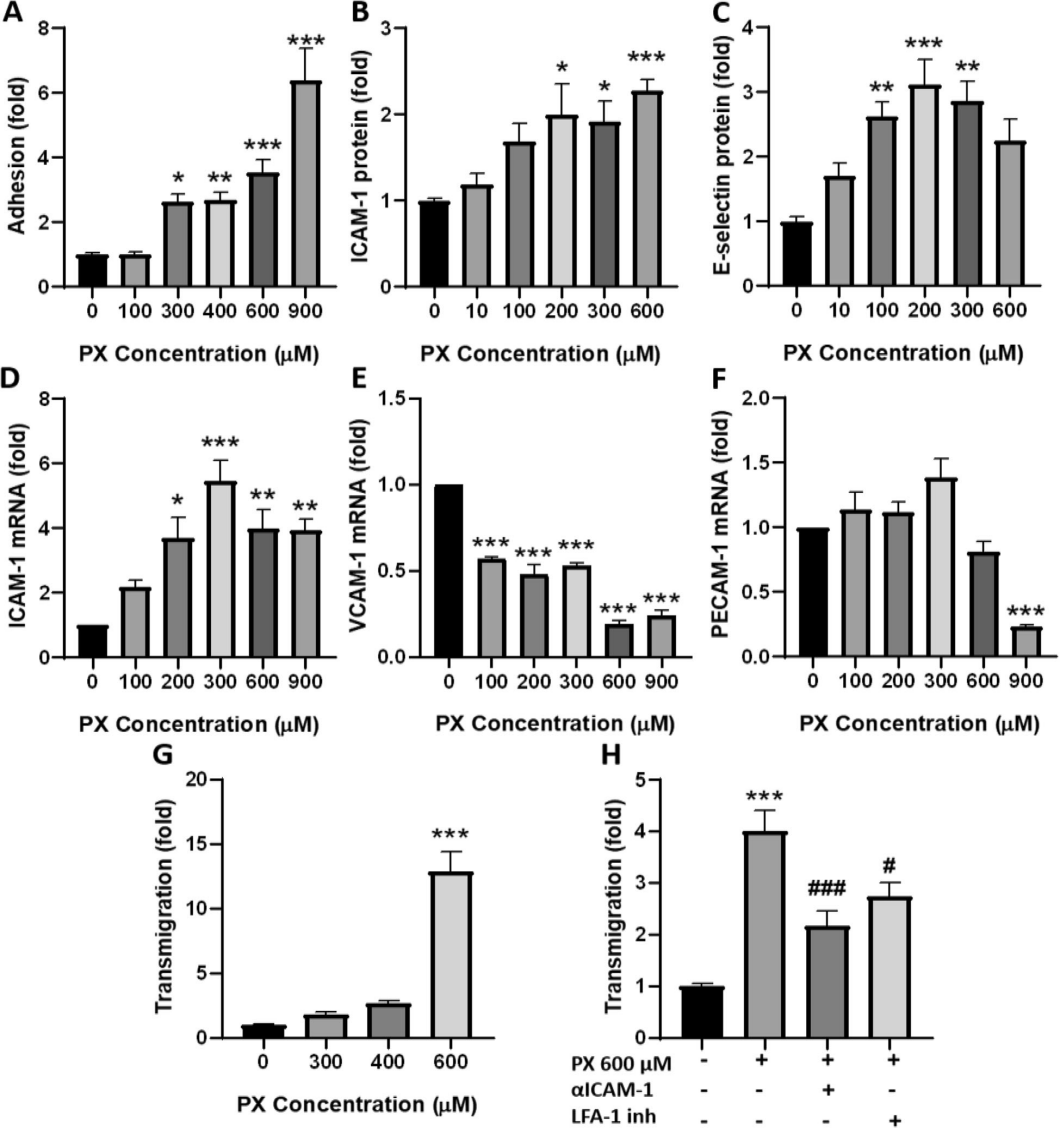
PBMCs adhesion and transmigration. **a** PX induced a dose-dependent increase in PBMCs adhesion to BLECs monolayers. *N* = 10-14 wells per treatment, from three independent experiments. **b-c** Protein expression level of ICAM-1 and E-selectin was assessed after 24 h treatment with PX, using in cell western blot assay. For ICAM-1: *N* = 11-14 wells per treatment, from three independent experiments. E-selectin: *N* = 6 from a single experiment. **d-f** mRNA levels of the adhesion molecules were determined after 24 h treatment with PX, using RT-PCR. (*N* = 3 biological repeats with 3 technical repeats). **g** BLECs were treated with increasing concentrations of PX (24 h). Freshly isolated human PBMCs were fluorescently labeled and added to the luminal side and let to transmigrate for 4 h. *N* = 12 wells per treatment, from three independent experiments. **h** ICAM-1/CD54 neutralizing antibody (10 μg/ml) was added simultaneously with PX (600 μM, 24 h) to the transmigration assay. For LFA-1 inhibition, PBMCs were pre-incubated with LFA-1 antagonist (5 μM, for 45 min prior to addition of PBMCs). *N* = 8-10 wells per treatment, from two independent experiments. Data presented as means normalized to control ± SEM. *p < 0.05, **p < 0.01, and ***p < 0.001 vs. control, #*p* < 0.05 and ###*p* < 0.001 vs. PX

Immune cells extravasation into brain parenchyma through the BBB is a multistep process, in which they bind to cerebral ECs, migrate across the blood vessel walls and enter the CNS [9]. One of the first steps in this process is the interaction of adhesion molecules expressed on brain ECs with corresponding ligands on the immune cell surface. Freshly isolated human PBMCs were added to the PX-treated monolayers. Only PBMCs that adhered firmly to BLECs were quantified. Figure 1a shows that treatment with PX increased the adherence of PBMCs in a dose-dependent manner suggesting an activation of the BLECs under these conditions.

The early tethering and rolling of immune cells on the endothelial surface are mediated by selectins, while the stronger attachment is dependent upon the recognition of ICAM-1 or VCAM-1 by the integrins expressed on the immune cells. Therefore, we examined the protein expression level of the adhesion molecules ICAM-1 (Fig. 1b) and E-selectin (Fig. 1c), and found that the expression level of ICAM-1 and E-selectin increased significantly along with increasing concentrations of PX. These results validate the existence of a multistep interaction process between the PBMCs and the endothelium upon treatment with PX.

Next, we examined if the expression of the adhesion molecules ICAM-1, VCAM-1, and PECAM-1 is modulated already at the transcriptional level. PECAM-1\CD31 is a member of the immunoglobulin (Ig) superfamily of cell adhesion molecules. PECAM-1 is mainly confined to ECs junctions where it promotes immune cell attraction and transendothelial migration [36] and participates in regulation of BBB permeability [37, 38]. Indeed, ICAM-1, VCAM-1, and PECAM-1 mRNA levels were differentially modified after treatment with PX. ICAM-1 mRNA levels increased significantly with the increasing concentrations of PX reaching a peak at 300 μM (Fig. 1d). On the other hand, VCAM-1 mRNA expression was reduced at all PX concentrations examined (Fig. 1e) and PECAM-1 mRNA levels decreased significantly only at the highest concentration tested (Fig. 1f).

The firm adherence of circulating PBMCs to inflamed vascular endothelium is an essential component of a multistep cascade that results in the accumulation and eventual migration through the vessel wall [9]. Therefore, subsequently to the adhesion, we examined the transmigration of PBMCs through the in vitro BBB model and revealed a dose-dependent increase in PBMCs transmigration starting with a 2.7 *±* 1.1 fold increase at 400 μM PX (which did not reach statistical significance) and 12.9 ± 1.1 fold increase at 600 μM PX (Fig. 1g).

PBMCs transmigration through the endothelium requires sequential interaction of adhesion molecules on the endothelial cell surface with corresponding ligands expressed on the immune cells. Lymphocyte function-associated antigen 1 (LFA-1) is a leukocyte cell surface glycoprotein that promotes adhesion in immunological and inflammatory reactions by binding to ICAM-1 [39]. We treated the BLECs with ICAM-1/CD54 neutralizing antibody simultaneously with PX or pre-incubated the PBMCs with LFA-1 antagonist, to evaluate if and how their interaction influence PX effects on PBMCs transmigration across the barrier. Figure 1h shows that both inhibitors significantly reduced the transmigration of PBMCs, strengthening their role in this process. Notably, fold change magnitude of PBMCs transmigration differs between Fig. 1h and g because different potencies of PX exist between batches and variance in the transmigration of PBMCs exist between experiments in which PBMCs were derived from different donors.

### Increased cell death by PX is not accountable for the enhanced transmigration

**Fig. 2:**
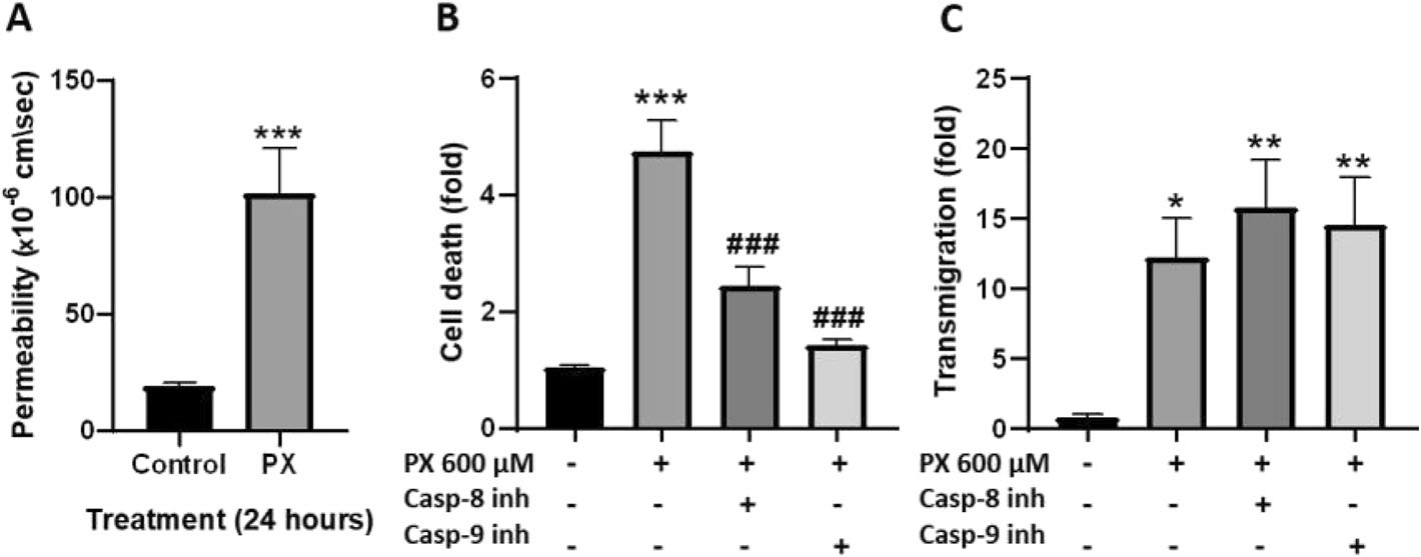
PX-induced transmigration is cell death independent. **a** Treatment of the BBB model with PX (600 μM, 24 h) increased the permeability to NaF. *N* = 9 from three independent experiments. Data presented as means of permeability coefficient (cm/s) ± SEM. ***p < 0.001 vs. control. **b** Medium from the treated luminal side was measured for cell death, using LDH release assay. *N* = 8-10 from two independent experiments (data obtained from the transmigration experiment presented in C). **c** Inhibition of caspases attenuated cell death but did not prevent PX-induced transmigration. Caspase-8 inhibitor or caspase-9 inhibitor (both at concentration of 150 μM) were added simultaneously with PX (600 μM, 24 h) to the transmigration assay. Data presented as means normalized to control ± SEM. *p < 0.05, **p < 0.01, and ***p < 0.001 vs. control and ###*p* < 0.001 vs. PX

Transmigration of immune cells across the endothelium barrier is a dynamic process that requires active interaction between the different cells and involves various molecular components. Paracellular leakage through gaps produced by cell death cannot account solely for a significant increase in PBMCs transmigration across the BBB but it can facilitate the process in some cases [40]. Therefore, we measured the effect of PX on the paracellular barrier properties by examining the permeability of the co-culture BBB model to the small non-permeable molecule sodium fluorescein (NaF, MW 376). Figure 2a shows that treating the BBB model with PX increased the permeability by 5.37 ± 0.09 fold.

To verify that the increased transmigration is indeed due to a PX-induced inflammatory response, and not simply due to increased cell death creating gaps in the confluent monolayer, we inhibited apoptosis using two known inhibitors for caspase-8 or caspase-9. Inhibition of these caspases would block two main apoptotic pathways: extrinsic pathway and intrinsic pathway, respectively [41]. Caspase-8 and caspase-9 inhibitors significantly rescued the PX-induced cell death (by 62.3% and 89.6%, respectively), yet had no effect on the PBMCs transmigration induced by PX (Fig. 2b and c). These results suggest that inhibiting cell death per se and preventing the opening of gaps created by dying cells, is not sufficient for reducing PBMCs transmigration across the barrier. Moreover, previous work in our lab demonstrated cell area enlargement of BLECs as a compensatory mechanism to maintain barrier function after exposure to PX [26]. The calculated cell area enlargement reached a maximum at 600 μM PX, meaning that there is a dynamic effort of the cells to seal the gaps and keep the monolayer intact, yet not sufficient to prevent transmigration. This strengthens the notion that the observed PBMCs transmigration is an active process evoked by pro-inflammatory stimuli and is not a passive route of immune cells crossing between adjacent ECs.

#### Activation of caspase-1

**Fig. 3:**
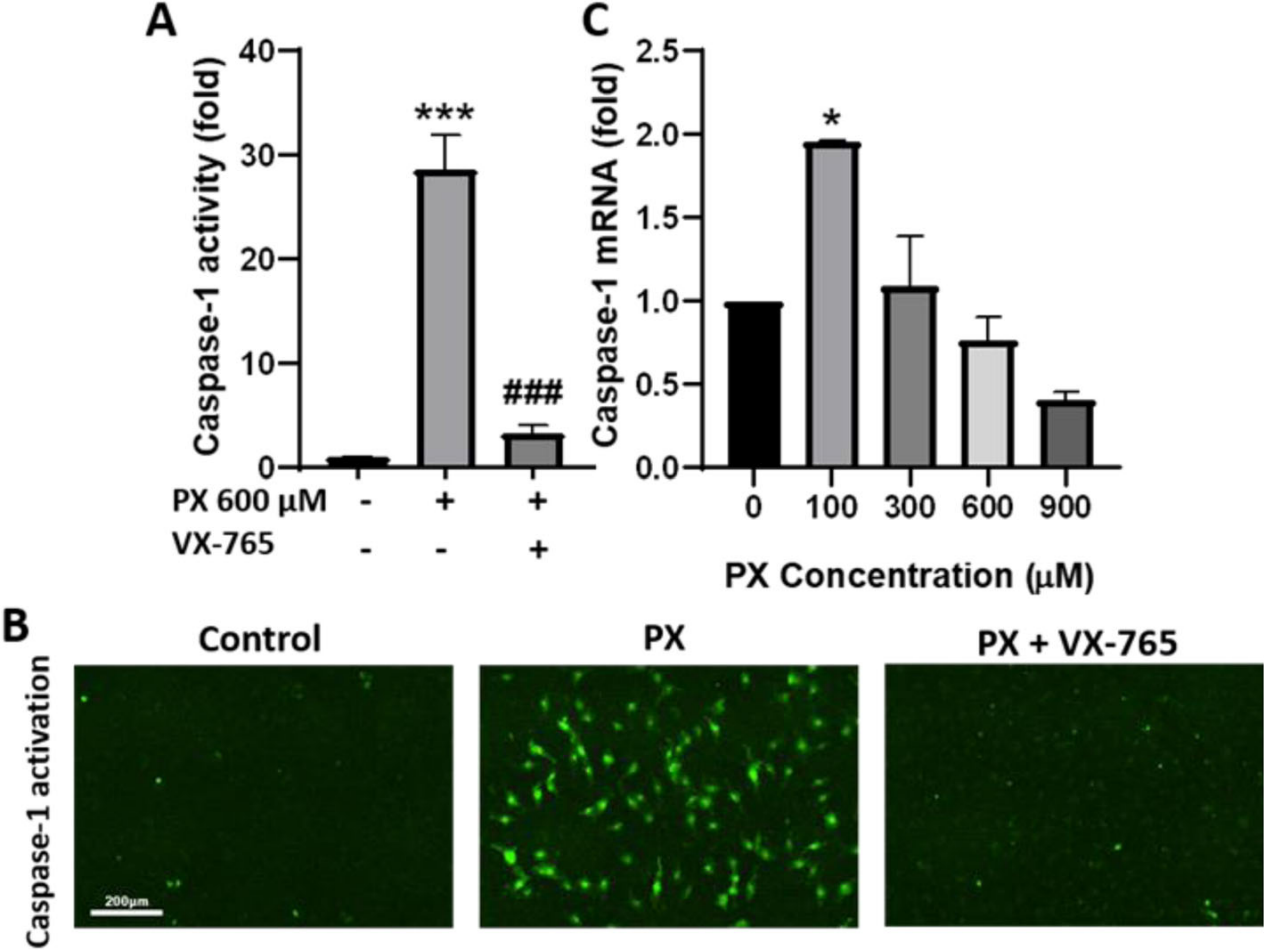
Activation of caspase-1 (**a**) Treatment of BLECs monolayers with PX (600 μM, 24 h) increased the activation of caspase-1, as assessed using fluorescent FLICA^Casp1^ probes (methods). *N* = 14-15 from three independent experiments. (**b**) Representative images showing increased caspase-1 activation in BLECs monolayers treated with PX (600 μM, 24 h) ± VX-765 (50 μM). Scale bar = 200 μm (**c**) mRNA levels of caspase-1 was determined after 24 h treatment with PX, using RT-PCR. *N* = 3 biological repeats with 3 technical repeats. Data are normalized to control and presented as means ± SEM. *p < 0.05, ***p < 0.001 vs. control and ###*p* < 0.001 vs. PX

In addition to the results obtained in Fig. 2, we have previously shown that blocking PX-induced apoptosis with the pan-caspase inhibitor ZVAD, failed to rescue barrier function disruption as manifested by increased permeability [26]. Therefore, we looked for a countermeasure compound that would integrate multiple molecular mechanisms. Caspase-1 plays a central role in cell immunity as an inflammatory response initiator besides the triggering of cell death and is involved in CNS disorders [20, 42]. We decided to examine its role in the endothelium function and inflammatory response. For that, we employed FLICA^Casp1^ probes, which are fluorescent-labeled inhibitors of caspase-1 that covalently bind to the active caspase enzyme [34]. Figure 3a and b shows that treating BLECs monolayers with PX increased the activation of caspase-1 by 28.6 ± 3.4 fold, and that this profound increase in caspase-1 activation is abolished with the addition of a caspase-1 specific inhibitor, VX-765. Figure 3c shows that caspase-1 mRNA levels were elevated at 100 μM PX and then declined with increasing concentrations of PX, possibly as a negative feedback response.

#### Inhibition of caspase-1 reduced adhesion and transmigration of PBMCs across the in vitro BBB model

**Fig. 4:**
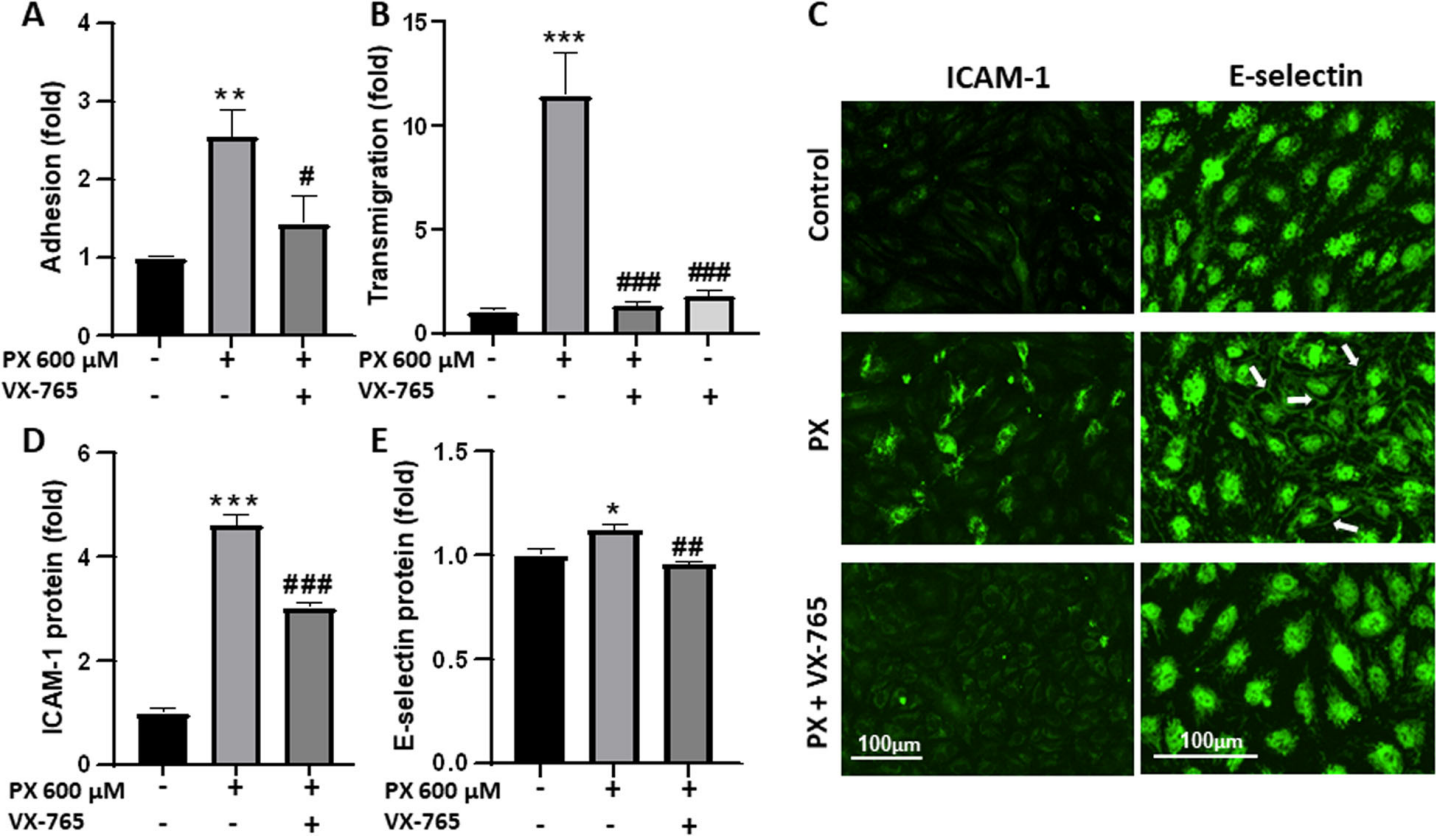
Caspase-1 inhibition reduced adhesion and transmigration of PBMCs across the in vitro BBB. VX-765 was added at concentration of 50 μM, simultaneously to treatment with PX (600 μM, 24 h). **a** VX-765 reduced the adhesion of PBMCs to BLECs monolayers. *N* = 10 from two independent experiments. **b** VX-765 reduced the transmigration of PBMCs through the in vitro BBB model. *N* = 8-11 from three independent experiments. **c** Representative images from immunofluorescence staining of ICAM-1 and E-selectin in BLECs monolayer. Arrows point to the increased E-selectin expression at the cell-cell junctions. Scale bar = 100 μm. **d-e** Quantification of ICAM-1 and E-selectin expression levels after treatment with PX ± VX-765. *N* = 6-8 from two independent experiments. Data presented as means normalized to control ± SEM. *p < 0.05, **p < 0.01 and ***p < 0.001 vs. control, #*p* < 0.05, ##*p* < 0.01, and ###*p* < 0.001 vs. PX

Next, VX-765 was utilized to assess caspase-1 role in PX-induced inflammatory response in the BBB. First, we demonstrated that VX-765 inhibited the adhesion of PBMCs to BLECs monolayers (Fig. 4a). Second, VX-765 reversed the transmigration of PBMCs across the BBB model back to control levels (Fig. 4b), and third, VX-765 attenuated the PX-induced increase in expression of the adhesion molecules ICAM-1 and E-selectin (Fig. 4c, d, and e). The images shown in Fig. 4c represent the increase in ICAM-1 staining, and an increased localization of E-selectin in cell-cell junctions (arrows), and demonstrate that these effects are attenuated with the inhibition of caspase-1. These multifaceted effects demonstrate that caspase-1 has a key role in triggering the immune response in BLECs following PX exposure.

**Fig. 5:**
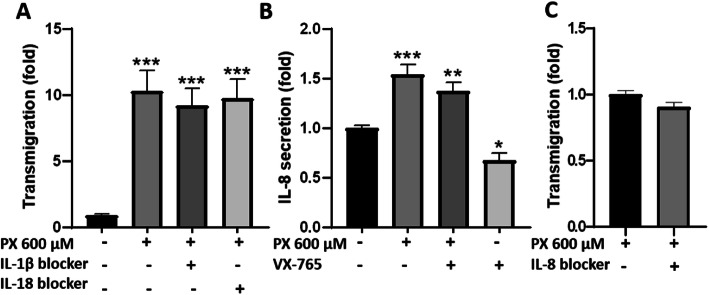
Role of IL-1β, IL-18, and IL-8 in PX-induced PBMCs transmigration. **a** IL-1β and IL-18 neutralizing antibodies (2 μg/ml) were added simultaneously to treatment with PX (600 μM, 24 h) to examine their effect on PBMCs transmigration across the BBB model. *N* = 12 wells per treatment, from three independent experiments. **b** IL-8 secretion from the BBB model was measured in media collected from the luminal side using an ELISA kit. *N* = 13 wells per treatment, from three independent experiments. Data presented as means normalized to control ± SEM. *p < 0.05, **p < 0.01, and ***p < 0.001 vs. control. **c** IL-8 neutralizing antibody (2 μg/ml) was added simultaneously to treatment with PX (600 μM, 24 h) to examine its effect on PBMCs transmigration. Data presented as means normalized to PX ± SEM. *N* = 6-11 wells per treatment, from two independent experiments

Caspase-1 cleaves the precursors of the inflammatory cytokines IL-1β and IL-18 and activates them [43]. Thus, we attempted to inhibit PX-induced PBMCs transmigration by neutralizing these downstream pro-inflammatory cytokines. Targeting IL-1β and IL-18 with neutralizing antibodies had no significant effect on PX-induced PBMCs transmigration (Fig. 5a). The chemokine IL-8 was shown to be involved in inflammatory processes and to have a role in promoting immune cell accumulation and transmigration to inflamed tissues [44] and its production in some instances was shown to be caspase-1-dependent [45]. Therefore, we examined its secretion from BBB models exposed to PX, and found significant increased levels. VX-765 reduced IL-8 levels in control cells by 33% suggesting partial involvement of caspase-1 in IL-8 secretion at the basal level in our model. However, there was no significant blocking effect for VX-765 when PX was added to the cells indicating a lack or limited role for caspase-1 in PX-induced endothelial cells activation (Fig. 5b). Similar to the results with IL-1β and IL-18, blocking IL-8 in the transmigration assay did not have significant effect on PX-induced PBMCs transmigration (Fig. 5c). Since no effect was obtained with the addition of these neutralizing antibodies, we verified their functionality and found that recombinant human IL-1β induced significant PBMCs adhesion to BLECs which was blocked by its antibody. Recombinant human IL-18 increased the adhesion of PBMCs by 37% (not statistically significant) and its antibody abolished this increase (Fig. S2, supplementary). Thus, blocking IL-1β or IL-18 is not sufficient to prevent PX-induced PBMCs transmigration in our PX/BBB model suggesting that other molecular factors should be targeted.

#### Inhibition of caspase-1 restored BBB integrity

**Fig. 6:**
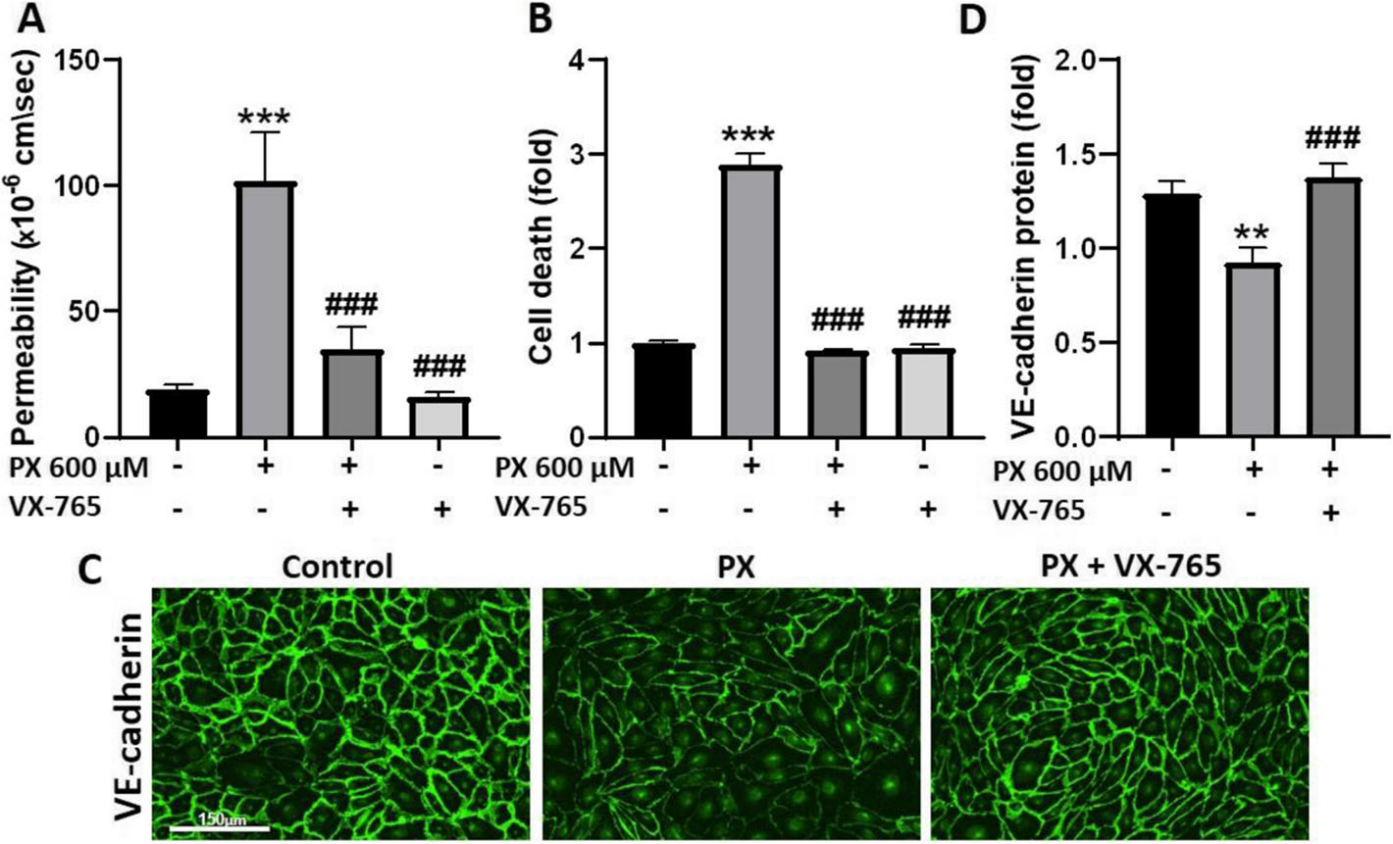
Inhibition of caspase-1 restored BBB integrity and VE-cadherin levels. VX-765 (50 μM) was added simultaneously to treatment with PX (600 μM, 24 h). **a** VX-765 blocked the PX-induced increase in permeability to NaF. *N* = 9 from three independent experiments (data taken from experiments also presented in Fig. 2a). Data presented as means of permeability coefficient (cm/s) ± SEM. **b** Medium from the treated luminal side was measured for cell death, using LDH release assay. VX-765 restored PX-induced cell death back to control levels. *N* = 8-10 from two independent experiments. **c** Representative images from immunofluorescence staining of VE-cadherin. Scale bar = 150 μm. **d** Quantification of VE-cadherin in BLECs monolayers demonstrated that VX-765 prevented the decrease in VE-cadherin expression. *N* = 8 from two independent experiments. Data presented as means normalized to control ± SEM. **p < 0.01, ***p < 0.001 vs. control, and ###*p* < 0.001 vs. PX

Caspase-1 is a key molecule composing the inflammasome complex. Activation of the inflammasome in ECs was shown to modulate inter-endothelial junction proteins and this was accompanied by enhanced transmigration of immune cells [24]. Here, we examined whether caspase-1 activation by PX causes disassembly of junctional proteins in vitro and by that may enhance paracellular transmigration of immune cells. As seen in Fig. 6a, inhibition of caspase-1 significantly restored the integrity of the barrier; the addition of VX-765 reduced the permeability by 80.3% compared to PX treatment. VX-765 rescued PX-induced cell death as well and restored it back to control levels (Fig. 6b). Permeability repair can be explained by the phenotypic rescue of the adherens junction (AJ) protein VE-cadherin achieved with the addition of caspase-1 inhibitor (Fig. 6c and d).

**Fig. 7:**
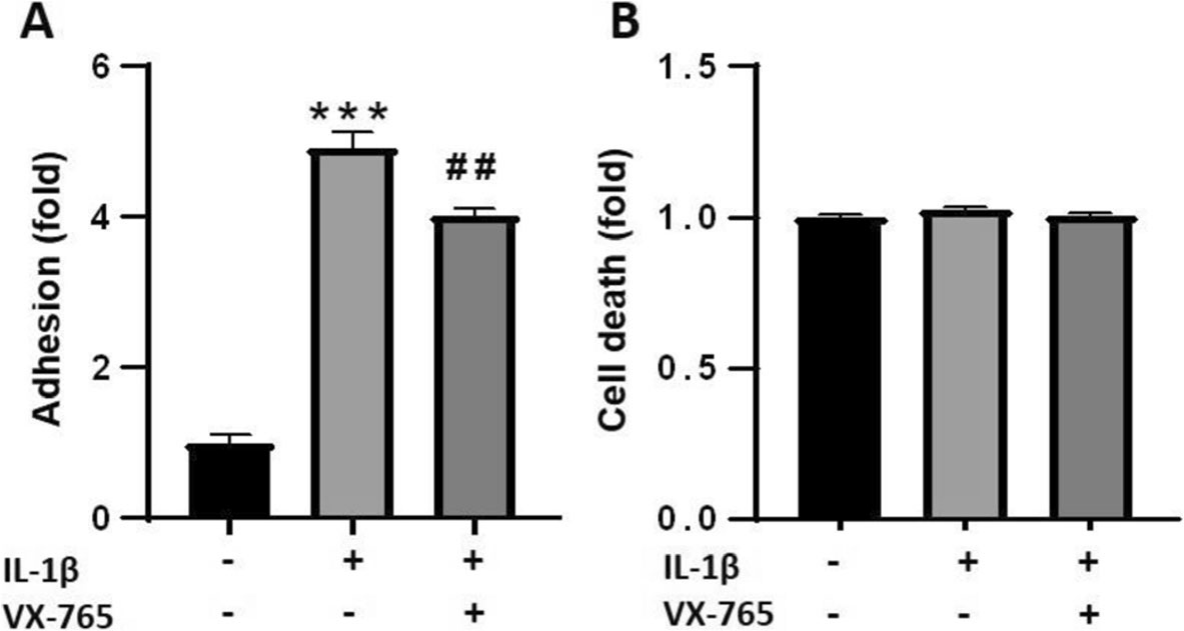
Caspase-1 inhibition reduced IL-1β-induced adhesion in non-toxic inflammatory conditions. VX-765 was added at concentration of 50 μM, simultaneously to treatment with IL-1β (10 ng/ml, 4 h). **a** VX-765 reduced the adhesion of PBMCs to BLECs monolayers. *N* = 10 from two independent experiments. **b** Medium from the treated BLECs was measured for cell death, using LDH release assay. *N* = 5 from a single experiment. Data presented as means normalized to control ± SEM. ***p < 0.001 vs. control and ##*p* < 0.01 vs. IL-1β

Since caspase-1 inhibition completely eliminated cell death, we wished to verify that indeed it is a key enzyme mediating inflammatory response in BLECs regardless of its role in regulating cell death. Therefore, we tested the effect of caspase-1 inhibition on adhesion of PBMCs to BLECs monolayer stimulated with IL-1β, caspase-1-downstream pro-inflammatory cytokine, and a well-established inflammatory response inducer [46]. IL-1β increased the adhesion of PBMCs to BLECs monolayers (Fig. 7a) and had no effect on cell death (Fig. 7b). Addition of VX-765 attenuated the increased adhesion, demonstrating that inhibition of caspase-1 possesses rescuing effect also in non-toxic inflammatory conditions.

#### Alterations of key BBB genes in vivo

**Fig. 8:**
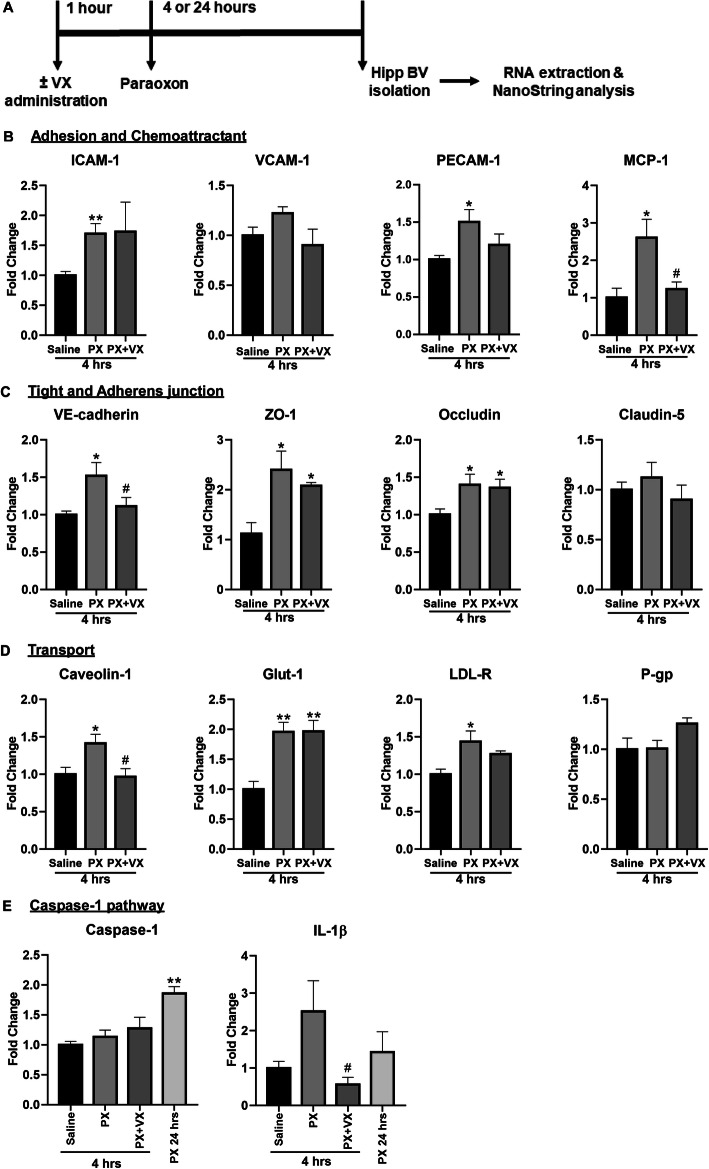
BBB gene levels after exposure to PX in vivo. (**a**) Timeline of the experiment. Mice were injected intramuscularly with saline or 0.45 mg\kg PX alone or 0.45 mg\kg PX pre-treated with 100 mg\kg VX-765 which was administrated IP, and their hippocampi were dissected 4 or 24 h later for blood vessels isolation and RNA extraction (all treatments were given for 4 h, unless mentioned differently). Fold change in mRNA expression levels of the indicated genes as determined by NanoString analysis: (**b**) Adhesion molecules and chemoattractant genes, (**c**) tight and adherens junction genes, (**d**) transporters related genes, (**e**) caspase-1 pathway genes. Fold change of each gene was calculated as the ratio of the average gene expression in the control group to that of the PX-treated groups. Normalized NanoString gene expression data are presented as mean fold change ± SEM. *N* = 4 mice for each treatment. *p < 0.05, **p < 0.01, ***p < 0.001 vs. control, and #*p* < 0.05 vs. PX

Mice were injected with saline or PX (0.45 mg\kg), with or without pre-administration of VX-765 (100 mg\kg). NanoString nCounter-based in vivo transcription analysis was performed on blood vessels isolated from hippocampi (Fig. 8a).

First, we confirmed a pro-inflammatory response by examining the modulation of cell adhesion molecules and chemoattractant genes expression following exposure to PX. Among these genes, ICAM-1 and PECAM-1 were significantly upregulated and VCAM-1 was upregulated but did not reach statistical significance (Fig. 8). PECAM-1 was reduced for some extent by addition of VX-765 as evident by the loss of statistical significance when compared to control. Members of the monocyte chemotactic protein (MCP)-1 family are considered the principal chemokines involved in the recruitment of monocytes/macrophages and activated lymphocytes [47]. In concordance with our in vitro transmigration results (Fig. 4b), MCP-1 levels were upregulated in response to PX and this effect was counteracted by the addition of VX-765 (Fig. 8b).

Next, tight and adherens junction genes, such as VE-cadherin, ZO-1 and occludin, were significantly upregulated 4 h post treatment with PX (Fig. 8c), implying that as an initial response, there is an induction of typical TJ and AJ gene expressions aiming to reestablish barrier properties [48]. Claudin-5 levels were similar in all groups. VX-765 administration significantly blocked the increase in VE-cadherin levels.

In addition, treatment with PX, induced alterations in genes of pivotal BBB transporters and endocytosis related genes. A significant increase in the mRNA levels of caveolin-1, Glut-1, and LDL-R was generated (Fig. 8d), suggesting that the BBB response involves also mechanisms of cell transport, metabolism and signal transduction. VX-765 administration completely blocked the increase in caveolin-1 levels and partially blocked the increase in LDL-R. P-glycoprotein mRNA levels remained the same after PX treatment.

Finally, an increase in mRNA levels of caspase-1 was measured 24 h after treatment with PX. The levels of its downstream substrate, IL-1β, were elevated which was blocked by VX-765 (Fig. 8e), further supporting our conclusion about the crucial involvement of the caspase-1 pathway in mediating the inflammatory response leading to BBB disruption.

## Discussion

In our last study, we demonstrated that PX was able to affect the permeability of a human in vitro BBB model and to modify TJ functioning [26]. The aim of this present study was to gain insight on the molecular processes underlying BBB inflammatory response to chemical exposure and to find potential therapeutic targets. The contribution of cerebral endothelial inflammasomes to neuroinflammation is presently unclear. We herein propose a mechanism that relies on caspase-1 activation that conveys a pro-inflammatory environment and a reduction in barrier integrity triggering transendothelial migration of PBMCs through the BBB. Adding PX to our system resulted in caspase-1 dependent adhesion of PBMCs to the BBB, as well as induction of adhesion molecules and the subsequent transmigration, which was partially dependent on ICAM-1—LFA-1 interaction. Noteworthy, barrier integrity was also compromised by PX and was rescued entirely by inhibiting caspase-1. Blocking apoptosis specifically by caspase-8 or 9 did not reduce transmigration levels. This was paralleled by a caspase-1 dependent blockade of decreased VE-cadherin protein expression explaining the rescue of barrier integrity by caspase-1 inhibition. However, neutralization of the known inflammasome/caspase-1 activated cytokines IL-18 or IL-1β did not influence PBMCs transmigration through the barrier. These findings were strengthened by in vivo experiments showing significant changes in transcription of junctional molecules, adhesion molecules, and inflammasome pathway components in hippocampal blood vessels from PX and VX-765 treated mice.

The enhanced transmigration observed (Fig. 1g and h) in the BBB in vitro model was dependent on ICAM-1—LFA-1 interaction, at least partially. We show that PX increased the mRNA and protein expression of the adhesion molecule ICAM-1 (Fig. 1d and b). ICAM-1 is a member of the immunoglobulin gene superfamily that is associated with ECs, and includes VCAM-1 and PECAM-1, as well. ICAM-1 is upregulated in various inflammatory conditions [49] and its upregulation supports our hypothesis of inflammatory like-phenotype induced in the endothelium. These results imply that ICAM-1 could be responsible for the adhesion and subsequent transmigration of PBMCs to and across the BBB. In contrast to the increase in ICAM-1, we found that VCAM-1 mRNA levels are downregulated (Fig. 1e). It is well established that ICAM-1, VCAM-1, and PECAM-1, have distinct roles in recruitment of immune cells. In various inflammatory diseases, VCAM-1 was shown to be regulated and expressed differently than ICAM-1, suggesting that distinct patterns of adhesion molecules expression are responsible for recruitment of different subtypes of leukocytes [50]. The blockade of ICAM-1 interactions with its ligand LFA-1, is a potential target for immunosuppression in our system, and its relevance as adhesion-based therapeutic strategy has been shown in various inflammatory conditions such as in autoimmune diseases and organ transplantation [49, 51].

Anti-PECAM-1 reagents were able to block leukocyte transmigration in mice [52], hence, increased PECAM-1 expression may facilitate immune cell extravasation [53]. These latter findings are in accordance with our in vivo results (Fig. 8b) showing enhanced gene expression of PECAM-1 by PX, which may reflect a more physiological early response than those observed under culture conditions, and its reduction by caspase-1 inhibition suggests it might be regulated by the inflammasome machinery.

We have previously shown that TJ gene levels are increased or decreased in brain ECs depending on the concentration of PX [26]. Assuming that a concentration gradient can be paralleled with duration of exposure, this might suggest a temporal compensatory mechanism of maintaining barrier properties induced at the beginning of exposure. Here, VE-cadherin, ZO-1, and Occludin mRNA levels were elevated in vivo 4 h post PX exposure (Fig. 8c). Importantly, we show that TJ and adherens protein levels are downregulated by PX in vitro (Fig. 6c and [26]) inferring their transcripts upregulation is a feedback response to their protein levels reduction. In line with these observations, the upregulation of TJ mRNA in brain endothelial cells was correlated with BBB disruption and a repair mechanism after exposure to TiO_2_ nanoparticles [48].

Various factors operate in the initiation and propagation of leukocytes extravasation across the BBB [9]. Disturbance of the TJ complexes between brain ECs leads to increased paracellular permeability, allowing leukocyte entry into inflamed brain tissue [54]. In the following section, we discuss possible inflammatory mechanisms leading to the propagation of leukocyte transmigration facilitated by impaired integrity of the BBB observed in our study.

There is accumulating evidence indicating the ability of cytokines to increase BBB permeability, by directly acting on the endothelium. The gene expression of the chemokine MCP-1 was upregulated in vivo by PX and this induction was dependent on caspase-1 activation (Fig. 8b). A significant increase in BBB permeability was observed during prolonged exposure to MCP-1 in vivo [55]. MCP-1 exerts its function by alternating TJ proteins distribution into a cytoplasmic compartment, suggesting that, besides its main function of recruiting leukocytes at sites of inflammation, MCP-1 also plays a role in “opening” the BBB, in accordance with our results (Fig. 6a). Stamatovic et al. demonstrated in vitro that MCP-1 induces TJ disassembly leading to an increase in permeability, through the internalization of claudin-5 and occludin via caveolae [54]. Our in vivo results show an increase in caveolin-1 mRNA levels after PX exposure which is abolished by caspase-1 inhibition (Fig. 8d). Singh and colleagues revealed that increase in caspase-1 activity is accompanied by elevation of lipid raft proteins such as caveolin-1 and an induction of lipid raft assembly. Lipid rafts are cholesterol-rich membrane microdomains well known to act as harbors for signaling molecules. They demonstrated the recruitment of proteins from the NLRP family in lipid rafts as well as their interaction with caveolin-1 [56]. Altogether, these data may suggest that upon PX exposure an inflammasome dependent pro-inflammatory response is initiated, which in turn induces the internalization of TJ proteins via caveolae.

Once activated through formation of an inflammasome complex, caspase-1 initiates a pro-inflammatory response, which results in the activation of the inflammatory cytokines: IL-1β and IL-18 [43]. We also show that the induction of the IL-1β gene in vivo by PX is caspase-1 dependent (Fig. 8e). Therefore, it is plausible that IL-1β mediates the effect of inflammation on integrity of the BBB in our system as was previously reported [57]. In addition, it was found that in ECs IL-18 increases the expression of matrix metalloproteinases (MMPs) [58], proteases that were found to degrade junctional proteins [59]. Infiltrating T lymphocytes appear to be the main source of MMP-2 and MMP-9, though brain ECs and pericytes also make an active contribution to MMP-9 production [60, 61]. Notably, MMP-9 gene expression was not elevated in our in vivo experiment (Fig. S3, Supplementary) but other members of the MMP family could have been involved.

IL-8 is a chemokine, which was found to be an important modulator of monocyte-endothelial interactions [44]. Because the production of IL-8 could be caspase-1/IL-1β-dependent [45], the effect of caspase-1 inhibition on IL-8 secretion was also assessed. Indeed, PX treatment increased the secretion of IL-8 from BLECs, but this secretion was not inhibited by VX-765 (Fig. 5b). Similar to neutralization of IL-1β or IL-18, treatment with IL-8 neutralizing antibody did not attenuate PX-induced PBMCs migration (Fig. 5a and c). It appears that several chemokines are actively participating in the recruitment of leukocytes to sites of cerebral inflammation. Thus, they may serve as targets for therapeutic intervention. However, aiming at leading chemokine family members is likely to have limited therapeutic application as there is considerable redundancy of function within this family [9].

Gene expression regulation of a few BBB transport-related genes was also influenced by PX as seen in our in vivo results. Glut-1 mRNA was elevated in vivo by PX and the same effect was observed in vitro (Fig. 8d and [26]). This implies a higher brain energy is demanded upon OPs damage, therefore, the BBB responds in elevating Glut-1 levels. A previously reported study showed that TNFα increases the abundance of Glut-1 transcripts in brain ECs [62], suggesting that inflammation may be the underlying cause for Glut-1 mRNA induction. However, caspase-1 inhibition was not able to reduce this induction in our in vivo setup.

LDL-R gene expression was upregulated by PX and reduced by caspase-1 inhibition in vivo (Fig. 8d). LDL-R functions in endocytosis of cholesterol-rich LDL. Cholesterol accumulation has been shown to activate the NLRP3 inflammasome in myeloid cells [63]. This could suggest a mechanism by which PX upregulates LDL-R expression leading to cholesterol accumulation and inflammasome activation. Furthermore, oxidized LDL upregulates PECAM-1 and downregulates VE-cadherin on endothelial junctions, both of which could promote leukocyte entry [52], compatible with our results.

In our in vivo assay P-gp gene expression was not altered (Fig. 8d), although we have previously observed that its mRNA expression was modulated in vitro [26]. The literature documents rapid downregulation of P-gp function in isolated rat brain microvessels in response to immune stimuli [64]. Although P-gp gene expression was not changed in our experiment it is likely that its function is reduced at least temporarily or at later stages post exposure, since it was demonstrated that OPs pesticides and specifically parathion, from which PX is metabolized, inhibit human P-gp transport function in intact cells [65].

We demonstrated an increased paracellular permeability of the barrier after PX treatment (Fig. 2a), which probably stemmed, at least in part, from increased cell death of the BLECs at these concentrations (Fig. 2b). We wished to further explore the cellular interaction between apoptosis, permeability, and transmigration of PBMCs across the barrier. For that, we inhibited cell death in our transmigration assay using caspase-8 and caspase-9 inhibitors, targeting cell apoptosis that was formerly shown to be involved in PX-induced cell death in other cell types [66], as well as in our lab (not shown). Despite the significant reduction in cell death, PBMCs transmigration was not decreased (Fig. 2b). Several studies have identified novel roles for caspase-8 in modulating IL-1β and inflammation [67]. In our system, it could still be possible that caspase-8 affects IL-1β levels but since an inhibitor of IL-1β did not have any influence on transmigration through our BBB model (Fig. 5a,) this effect of caspase-8 is not discernible. All these findings support the notion that endothelial cell death and PBMCs transmigration are not necessarily interrelated and that PBMCs transmigration is an active process evoked by pro-inflammatory stimuli. These results imply that an appropriate therapeutic target for rescuing a complex damage to the BBB should involve the cell death axis, as well as the inflammatory axis. Indeed, caspase-1 is involved in both axes, but noteworthy; inhibition of caspase-1 can also attenuate the increased adhesion in non-toxic inflammatory conditions (Fig. 7a).

Caspase-1 mRNA was elevated at 24 h after PX injection in mice (Fig. 8e) and in vitro its mRNA level was elevated at 100 μM while at higher doses its expression started to decline (Fig. 3c). Importantly, at 600 μM caspase-1 protein activation was significantly induced in vitro, emphasizing the important role it plays in the BBB after PX exposure (Fig. 3a and b). Looking for a compound that will integrate both inhibition of cell death and inflammation properties, we used VX-765, a potent caspase-1 inhibitor that works efficiently on both pathways. In particular, VX-765 reduced PX-induced PBMCs adhesion to endothelial monolayer, completely abolished their migration across the BBB model (Fig. 4a and b), restored BLECs viability, as well as BBB integrity (Fig. 6a and b).

Caspase-1 activation functionally links vascular and neurological diseases and hence represents a promising therapeutic target [18]. Humans who possess genetic variants of caspase-1 that display low enzymatic activity do not suffer from immunodeficiency [68]. Furthermore, caspase-1 KO mice are fertile and healthy indicating that caspase-1 is not essential for proper development [69]. Induction of caspase-1 and activation of IL-1β both occur in human epilepsy, and contribute to experimentally induced acute seizures. VX-765 has been shown to reduce chronic epileptic activity and acute seizures in mice [70] and was found to be safe and well-tolerated in a phase II clinical trial in people with treatment-resistant epilepsy [71].

Several in vivo studies have shown that BBB permeability is increased after exposure to organophosphates [72] and specifically to PX [73]. The functional in vivo effects of inhibiting caspase-1 in this scenario should be further examined in future studies.

## Conclusions

Ideally, novel medical countermeasures would focus on developing therapeutic approaches that selectively interfere with those aspects of the inflammatory response that promote damage while protecting or promoting aspects of repair processes. We suggest the utilization of molecules that can inhibit caspase-1 as potential therapeutic candidates that may protect the brain from vascular insult and hence neuronal damage in a variety of CNS disorders where the BBB is inflamed**.**

## Supplementary information

**Additional file 1: Table S1.** In-vivo treatments.

**Additional file 2: Figure S1.** Ruling out PX-induced pH interference in BCECF-stained cells. Similar fluorescence values were obtained for different amount of BCECF-labeled PBMCs in control and PX (600 μM) containing media, directly loaded into the wells. The PX containing medium itself had no influence on fluorescence intensity, ruling out any PX-induced pH related artifact. Fluorescence detection was carried out with an Infinite 200 PRO (Tecan) plate reader using the excitation/emission wavelength settings: 485/538 nm. Data presented as mean fluorescence of two wells per treatment ± SEM. a.u, arbitrary unit.

**Additional file 3: Figure S2.** Role of IL-1β and IL-18 in PBMCs adhesion. BLECs were treated with IL-1β (10 ng/ml) or IL-18 (10 ng/ml), and the functionality of their neutralizing antibodies was examined. IL-1β and IL-18 neutralizing antibodies were added simultaneously with the cytokines treatment (2 μg/ml, 4 hrs). IL-1β evoked adhesion of PBMCs to BLECs monolayers and its neutralizing antibody abolished this effect. N = 5 from two independent experiments. Data presented as means normalized to Control ± SEM. ***p < 0.001 vs. control and ### < 0.001 vs. IL-1β.

**Additional file 4: Figure S3.** MMP-9 mRNA expression in blood vessels after exposure to PX. Mice were injected with saline or 0.45 mg\kg PX alone or 0.45 mg\kg PX pre-treated with 100 mg\kg VX-765, and their hippocampi were dissected 4- or 24-hrs later for blood vessels isolation and RNA extraction. Fold change in mRNA expression levels was calculated as the ratio of the average expression in the control group to that of the PX group, as determined by NanoString analysis. Normalized NanoString gene expression data are presented as mean ± SEM. N = 3-4 mice for each treatment. **p < 0.01 *vs*. control.

## Data Availability

The datasets used and/or analyzed during the current study are available from the corresponding author on reasonable request.

## Abbreviations

AJ: Adherens junction
AD: Alzheimer’s disease
BBB: Blood-brain barrier
BLECs: Brain-like endothelial cells
CNS: Central nervous system
ECs: Endothelial cells
ECM: Endothelial cell medium
ICAM-1: Intercellular adhesion molecule-1
IL: Interleukin
LFA-1: Lymphocyte function-associated antigen-1
MCP-1: Monocyte Chemoattractant Protein-1
NLRP: Nucleotide-binding oligomerization domain, leucine-rich repeat and pyrin domain containing
OPs: Organophosphates
PX: Paraoxon
PECAM-1: Platelet endothelial cell adhesion molecule-1
PBMC: Peripheral blood mononuclear cell
Pe: Permeability
TJ: Tight junction
VCAM-1: Vascular cell adhesion molecule-1
