# Supplementary material for: Caspase-1 has a critical role in blood-brain barrier injury and its inhibition contributes to multifaceted repair
Source: J Neuroinflammation. 2020 Sep 9;17:267. doi: 10.1186/s12974-020-01927-w (PMC7488082; doi:10.1186/s12974-020-01927-w)
Supplement: Supplementary file 1 — Additional file 1: Table S1. In-vivo treatments. [file 12974_2020_1927_MOESM1_ESM.docx]

| **Group** | | **t = - 1 hour** | **t = 0 min** | **t = 4 min** | **t = 4 hours** | **t = 24 hours** |
| --- | --- | --- | --- | --- | --- | --- |
| **1** | **Paraoxon**  **4 hrs** |  | Paraoxon  0.45 mg/kg (IM) | Atropine: 1.5 mg/kg (IP) | Hippocampus Dissection |  |
|  |  |  |  | Obidoxime: 20 mg/kg (IP) |  |  |
| **2** | **Paraoxon + VX-765** | VX-765  100mg/kg | Paraoxon  0.45 mg/kg (IM) | Atropine: 1.5 mg/kg (IP) | Hippocampus Dissection |  |
|  |  |  |  | Obidoxime: 20 mg/kg (IP) |  |  |
| **3** | **Control** |  | Saline (IM) |  | Hippocampus Dissection |  |
| **4** | **Paraoxon**  **24 hrs** |  | Paraoxon  0.45 mg/kg (IM) | Atropine: 1.5 mg/kg (IP) |  | Hippocampus Dissection |
|  |  |  |  | Obidoxime: 20 mg/kg (IP) |  |  |
