# Supplementary figures and images for: Caspase-1 has a critical role in blood-brain barrier injury and its inhibition contributes to multifaceted repair
Source: J Neuroinflammation. 2020 Sep 9;17:267. doi: 10.1186/s12974-020-01927-w (PMC7488082; doi:10.1186/s12974-020-01927-w)

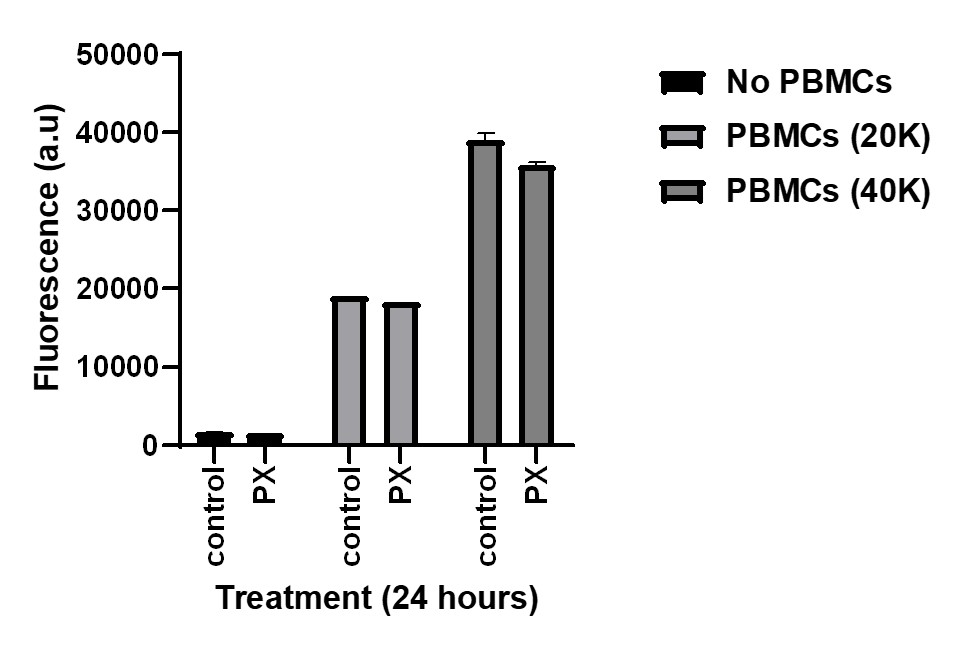

Supplement: Supplementary file 2 — Additional file 2: Figure S1. Ruling out PX-induced pH interference in BCECF-stained cells. Similar fluorescence values were obtained for different amount of BCECF-labeled PBMCs in control and PX (600 μM) containing media, directly loaded into the wells. The PX containing medium itself had no influence on fluorescence intensity, ruling out any PX-induced pH related artifact. Fluorescence detection was carried out with an Infinite 200 PRO (Tecan) plate reader using the excitation/emission wavelength settings: 485/538 nm. Data presented as mean fluorescence of two wells per treatment ± SEM. a.u, arbitrary unit. [file 12974_2020_1927_MOESM2_ESM.jpg]

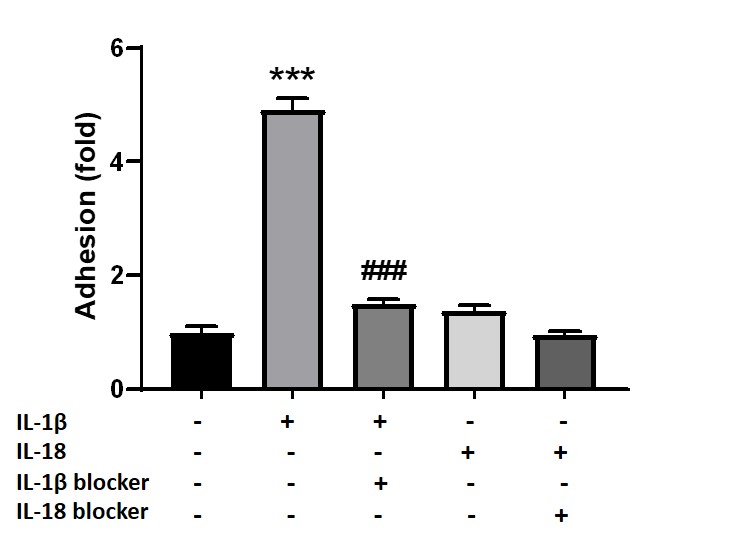

Supplement: Supplementary file 3 — Additional file 3: Figure S2. Role of IL-1β and IL-18 in PBMCs adhesion. BLECs were treated with IL-1β (10 ng/ml) or IL-18 (10 ng/ml), and the functionality of their neutralizing antibodies was examined. IL-1β and IL-18 neutralizing antibodies were added simultaneously with the cytokines treatment (2 μg/ml, 4 hrs). IL-1β evoked adhesion of PBMCs to BLECs monolayers and its neutralizing antibody abolished this effect. N = 5 from two independent experiments. Data presented as means normalized to Control ± SEM. ***p < 0.001 vs. control and ### < 0.001 vs. IL-1β. [file 12974_2020_1927_MOESM3_ESM.jpg]

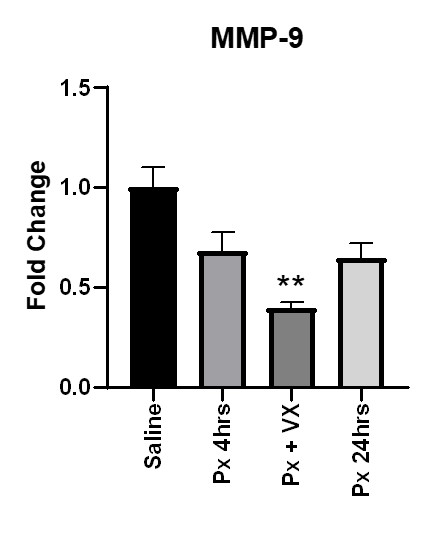

Supplement: Supplementary file 4 — Additional file 4: Figure S3. MMP-9 mRNA expression in blood vessels after exposure to PX. Mice were injected with saline or 0.45 mg\kg PX alone or 0.45 mg\kg PX pre-treated with 100 mg\kg VX-765, and their hippocampi were dissected 4- or 24-hrs later for blood vessels isolation and RNA extraction. Fold change in mRNA expression levels was calculated as the ratio of the average expression in the control group to that of the PX group, as determined by NanoString analysis. Normalized NanoString gene expression data are presented as mean ± SEM. N = 3-4 mice for each treatment. **p < 0.01 vs. control. [file 12974_2020_1927_MOESM4_ESM.jpg]
